# Construction and validation of novel nomograms based on the log odds of positive lymph nodes to predict the prognosis of papillary thyroid cancer: a retrospective cohort study

**DOI:** 10.3389/fendo.2025.1411426

**Published:** 2025-03-07

**Authors:** Saisai Jing, Jiazhao Song, Yupeng Di, Jiajia Xiao, Jianke Ma, Zimiao Wu

**Affiliations:** ^1^ Department of Oncology, Affiliated Cixi Hospital, Wenzhou Medical University, Cixi, Zhejiang, China; ^2^ Department of Radiotherapy, Air Force Medical Center, Air Force Medical University, Beijing, China; ^3^ Graduate School, Hebei North University, Zhangjiakou, Hebei, China

**Keywords:** papillary thyroid cancer, nomogram, SEER, post-operative, long-term overall survival

## Abstract

**Objective:**

This study aims to assess the long-term prognostic significance of the log odds of positive lymph nodes (LODDS) in patients diagnosed with papillary thyroid cancer (PTC) and to develop a novel nomogram for predicting long-term overall survival (OS).

**Methods:**

The cohort was randomly divided at a ratio of 7:3 from the Surveillance, Epidemiology, and End Results (SEER) database. Additionally, patient data from a medical center in China served as an external validation cohort. Nomograms were constructed using data from the training cohort and subsequently validated using both internal and external validation cohorts to predict 120- and 180-month OS in PTC patients. The predictive performance and clinical utility of the nomogram were assessed using various metrics, including the concordance index (C-index), time-dependent receiver operating characteristic (ROC) curves, calibration curves, decision curve analysis (DCA), Integrated Discriminant Improvement Index (IDI), and Net Reclassification Improvement Index (NRI).

**Results:**

LODDS is an independent prognostic factor for PTC, a nomogram demonstrating high accuracy in predicting long-term OS. The C-index values, and time-dependent area under the curve (AUC) indicated well discriminatory ability of the nomogram. Calibration plots exhibited high concordance, while DCA, NRI, and IDI analyses revealed superior performance of the nomogram compared to AJCC staging system.

**Conclusion:**

The clinical prediction model incorporating LODDS exhibits robust predictive performance, aiding in the assessment of long-term prognosis post-surgery in PTC patients. It serves as a valuable adjunct to the AJCC system, offering a scientific basis for guiding interventions and rehabilitation strategies for PTC patients following surgery.

## Introduction

Papillary thyroid cancer (PTC) is the most common thyroid malignancy, accounting for approximately 85% of cases (1). In the last 30 years, the incidence of PTC has continued to increase globally (2). Generally, the 5-year survival rate of PTC is higher than 95%, which has a better prognosis than other malignant tumors, but it should not be ignored that lymph node metastasis (LNM) still exists in 30%-80% of PTC patients (3), which affects the long-term prognostic situation. Therefore, exploring postoperative lymph nodes (LN) is of practical clinical significance for the long-term prognostic assessment and further treatment of PTC patients. Currently, variables related to the comprehensive assessment of lymph nodes, such as the total number of postoperative LN, the number of positive lymph nodes (PLN), and the area of lymph node metastasis, have been included in other studies related to the prognostic assessment of malignant tumors (4–6). However, N staging in existing the 8th edition of AJCC staging does not include the number of LN in the consideration of assessment (7), which may affect the accuracy of assessing the long-term prognosis of patients with PTC. Therefore, further studies may be needed to explore new lymph node staging and to further optimize the grading system for PTC.

As a class of scientific clinical prognostic assessment tools, nomogram have gradually become a common method to study the prognosis of patients with various types of cancers by visualizing the calculation and combining the prognostic factors to predict the survival rate scientifically (8–10). In recent years, several studies have developed nomograms based on clinical features, pathologic factors, and blood and imaging tests to assess the prognosis of patients with PTC (11–15). The predictive power of the prediction model was assessed by analyzing the receiver operating characteristic (ROC) curves, the area under the curve (AUC), concordance index (C-index), and decision curve analysis (DCA) curves of the nomogram to compare with the existing clinical staging systems. The nomogram constructed based on different prognostic factors demonstrated superior individualized survival prediction efficacy and clinical applicability.

Currently, new LN staging modalities, such as positive LN logarithmic ratio based on the log odds of positive lymph nodes (LODDS) or LN ratio (LNR). There have also received increasing attention, and several studies have demonstrated more accurate predictive ability than the traditional TNM staging system (16–18). However, relevant studies addressing the long-term prognostic value of new LN staging modalities in PTC are still lacking. Our study aimed to clarify whether the LODDS could be utilized to predict long-term overall survival (OS) in patients with PTC as a way to explore the feasibility of establishing a new LN staging system. On this basis, a new LN staging prognostic model based on nomogram was established by analyzing the relevant factors affecting the prognosis of postoperative PTC patients. Different prognostic risk groups were distinguished as a way to predict the long-term prognosis of PTC patients more accurately.

## Methods

### Patient sources and study design

This study was based on a multicenter retrospective cohort from the United States and China following the Transparent Reporting of Individual Prognostic or Diagnostic Multivariate Predictive Models (TRIPOD) (19). The modeled patient clinical data for this study were obtained from the Surveillance, Epidemiology, and End Results (SEER) 17 Registry Research Database in the U.S. Covering approximately 30% of the U.S. population, the SEER database includes many items such as patient demographics, tumor information, and follow-up status, providing a large sample of clinically valuable information for prognostic studies of tumors (20). Patient follow-up data from a medical center in China was used as an external validation cohort to verify the generalizability of the prediction model. Because the SEER database is publicly available and anonymous, the modeling data did not require institutional review board approval and individual patient consent applications. The Chinese dataset for this study was derived from clinical consultation data of PTC patients kept from January 1, 2000, to December 31, 2010, at Cixi Hospital of Wenzhou Medical University, Zhejiang Province, China. The establishment of this dataset was approved by the Ethics Committee of the hospital (No. 2023-LP-LW007), and the follow-up information was obtained by verbal communication confirmed by telephone, and the process of obtaining patient information followed the requirements of the Ethics Committee. This study complies with the Declaration of Helsinki (21).

### Clinical cohort screening and definitions

Patients with thyroid cancer diagnosed and treated between 2004 and 2015 (anatomical code: C73.9) with a follow-up date up to October 31, 2022, were selected according to the International Classification of Diseases in Oncology, version 2023 (ICD-O-3) codes in the SEER database and further screened. Inclusion criteria: 1. papillary cancer were selected as study subjects (histologic codes: 8040/3, 8042/3,8043/3,8044/3); 2. surgical procedure was localized thyroidectomy, thyroid isthmus resection, or more extensive (surgical codes: 20,21,22,23,26,27,30,40,50); and 3. patients who were non-dead or post-mortem post mortem diagnosis. Exclusion criteria: 1. patients younger than 18 years of age; 2. pathological confirmation not performed; 3. first primary site of the tumor was not the thyroid gland; 4. survival time after treatment was less than one month; 5. incomplete or unknown clinical information about the patient. Patients were randomly assigned according to a ratio of 7:3 to construct a training cohort and an internal validation cohort, respectively; the training cohort (n=2303) was used for prediction modeling, and the internal validation cohort (n=986) was used for internal validation of the prediction model. According to the recording criteria of the SEER database, the years 2004-2015 could correspond to the sixth or seventh edition of AJCC staging information. The study team reclassified the TNM classification according to the eighth edition. The external validation cohort (n=215) had a follow-up date up to December 31, 2023. Inclusion criteria for patients: 1. PTC confirmed by postoperative pathology. Exclusion criteria: 1. no pathologically confirmed diagnosis; 2. non-primary thyroid tumor; 3. lost to follow-up; 4. survival time <1 month after diagnosis; 5. unknown tumor TNM stage. Pathologic staging records for the external validation cohort were also based on the 8th edition of AJCC staging system.

Baseline demographic data from the SEER database included age, gender, and race. The baseline of tumor characteristics included tumor differentiation and TNM stage. Additionally, information regarding surgical interventions (local thyroidectomy/thyroidectomy isthmus, subtotal thyroidectomy, total thyroidectomy), radiotherapy, chemotherapy, and LN were extracted. LNR was defined as the ratio of the number of positive lymph nodes (PLN)/total number of removed lymph nodes (RLN). LODDS was defined as lg[(PLN + 0.50)/(negative lymph nodes (NLN) + 0.50)]. To avoid infinite numbers, 0.50 was added to both the numerator and denominator (16). OS in all cohorts was defined as the time interval from the date of diagnosis to death from any cause or the end of the last follow-up.

### Constructing and validating nomogram models

Lasso-multifactorial Cox regression analyses of all variables with *p* < 0.05 were further included in the construction of the final model, which was cross-validated 10 times to construct a nomogram composite scoring system for predicting the probability of overall survival. Internal validation was accomplished by taking a bootstrap procedure of 1000 resamples to the internal validation cohort. External validation was performed using the external validation cohort. Nomogram performance was assessed using subject ROC curves and calibration curves with AUC of ROC, ranging from 0.5 (no discrimination) to 1 (full discrimination). DCA was utilized to determine the predicted net benefit threshold. The constructed line plots were compared to the traditional AJCC/TNM staging by calculating the net reclassification improvement (NRI) and the integrated discrimination improvement (IDI), and the Z-test was used to test for differences.

After building the predictive model, the survival status, survival time, and linear predictors of the predictive model from the training cohort were selected separately and imported into the X-tiles software, and the linear predictors were used as markers for the analysis. Statistical analysis by the X-tiles software will result in a score against the linear predictors on the risk stratification for all predicted probability points calculated as a score based on the linear prediction of the patient’s nomogram.

### Statistical analysis

Descriptive analysis was performed for all patients. Categorical variables are expressed as numbers and percentages (%). Continuous variables conforming to normal distribution are expressed as mean ± standard deviation, and continuous variables conforming to skewed distribution are expressed as median and interquartile range. Categorical variables were compared using the χ2 test or Fisher’s exact test, and continuous variables were compared using the t-test. Hazard ratios (HR) and 95% confidence interval (CI) were recorded for the multifactor Cox proportional risk model. Survival curves were plotted using the Kaplan-Meier method and differences in survival between curves were analyzed using the log-rank test. The best cutoff point most relevant to clinical prognosis was determined by X-tile software. Prognostic risk stratification was also performed (22).

SEER patient data were extracted based on SEER * Stat 8.4.3 software. The collected data were fully statistically analyzed using R v.4.4.1 statistical software (http://www.R-project.org, R Foundation, Vienna, Austria) and Free Statistics Software version 1.9 statistical software (23). A two-tailed *p* < 0.05 for the final results was considered statistically significant.

## Results

### Training and validation cohort characteristics

**Figure 1:**
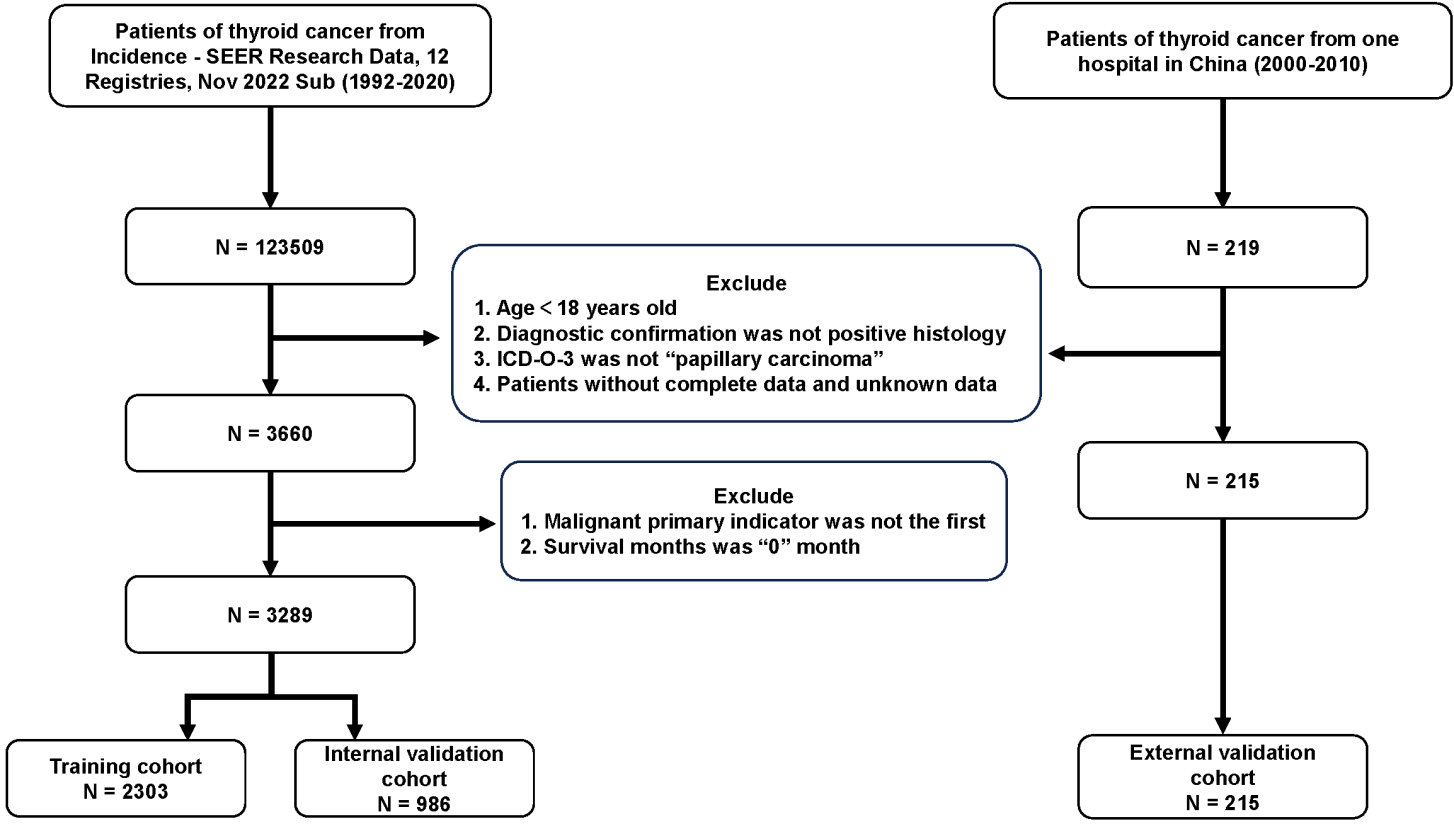
Flow chart for PTC patients. PTC, papillary thyroid cancer; SEER, the Surveillance, Epidemiology, and End Results; Sub, submission; ICD-O-3, International Classification of Diseases for Oncology, 3rd Edition.

A total of 3504 patients participated in the analysis, assessing the demographic and clinical characteristics of the different cohorts. Of these, 2303 belonged to the training cohort, 986 to the internal validation cohort, and 215 to the external validation cohort (**Figure 1**). In terms of age, young and middle-aged people predominated in the training cohort (1,558, 67.7%), while the mean age of the external validation cohort was 47.7 ± 12.8 years, with a predominance of young and middle-aged patients (145, 67.4%). In the distribution of tumor characteristics in the training cohort, highly differentiated tumors were present in 76.0% of patients. The number of patients with stage I was (155, 89.6%) who underwent total thyroidectomy. The distribution of patient tumor characteristics for the external validation cohort was similar to that of the training cohort. In the training cohort, the median RLN was 3.0 (IQR: 1.0-7.0), the median PLN was 0.0 (IQR: 0.0-1.0), the median NLN was 2.0 (IQR: 1.0-6.0), the median LNR was 0.0 (IQR: 0.0-2.0), the median RLN was 3.0 (IQR: 1.0-7.0), and the median LODDS was -0.1 (IQR: -0.2 -0.0). In the external validation cohort, the median LODDS was -0.5. No statistically significant differences were observed between the training cohort and the internal validation cohort (*p*-value > 0.05). Detailed information about the different cohort can be found in **Tables 1**, **2** respectively.

**Table 1:** Baseline characteristics of patients for training cohort and internal validation cohort from SEER database.

Variables	Total (n = 3289)	Training cohort (n = 2303)	Internal validation cohort (n = 986)	*p*-Value
Age				0.135
18-54	2245 (68.3)	1558 (67.7)	687 (69.7)	
55-66	636 (19.3)	442 (19.2)	194 (19.7)	
>66	408 (12.4)	303 (13.2)	105 (10.6)	
Sex				0.442
Male	698 (21.2)	497 (21.6)	201 (20.4)	
Female	2591 (78.8)	1806 (78.4)	785 (79.6)	
Race				0.593
White	2793 (84.9)	1953 (84.8)	840 (85.2)	
Black	169 (5.1)	124 (5.4)	45 (4.6)	
Other	327 (9.9)	226 (9.8)	101 (10.2)	
Grade				0.659
G1	2495 (75.9)	1750 (76)	745 (75.6)	
G2	603 (18.3)	420 (18.2)	183 (18.6)	
G3	152 (4.6)	109 (4.7)	43 (4.4)	
G4	39 (1.2)	24 (1.0)	15 (1.5)	
T				0.533
T1	1724 (52.4)	1204 (52.3)	520 (52.7)	
T2	631 (19.2)	454 (19.7)	177 (18.0)	
T3	745 (22.7)	510 (22.1)	235 (23.8)	
T4	189 (5.7)	135 (5.9)	54 (5.5)	
N				0.315
N0	2193 (66.7)	1548 (67.2)	645 (65.4)	
N1	1096 (33.3)	755 (32.8)	341 (34.6)	
M				0.679
M0	3224 (98.0)	2259 (98.1)	965 (97.9)	
M1	65 (2.0)	44 (1.9)	21 (2.1)	
Stage				0.516
I	2219 (67.5)	1550 (67.3)	669 (67.8)	
II	233 (7.1)	155 (6.7)	78 (7.9)	
III	490 (14.9)	352 (15.3)	138 (14.0)	
IV	347 (10.6)	246 (10.7)	101 (10.2)	
Surgery				0.207
Localized thyroidectomy/thyroid isthmus resection	292 (8.9)	192 (8.3)	100 (10.1)	
Subtotal thyroidectomy	70 (2.1)	47 (2.0)	23 (2.3)	
Total thyroidectomy	2927 (89.0)	2064 (89.6)	863 (87.5)	
RLN				
Median (IQR)	3.0 (1.0, 7.0)	3.0 (1.0, 7.0)	3.0 (1.0, 7.0)	0.764
PLN				
Median (IQR)	0.0 (0.0, 1.0)	0.0 (0.0, 1.0)	0.0 (0.0, 1.0)	0.261
NLN				
Median (IQR)	2.0 (1.0, 6.0)	2.0 (1.0, 6.0)	2.0 (1.0, 5.0)	0.280
LNR				
Median (IQR)	0.0 (0.0, 0.2)	0.0 (0.0, 0.2)	0.0 (0.0, 0.3)	0.226
LODDS				
Median (IQR)	-0.1 (-0.2, 0.0)	-0.1 (-0.2, 0.0)	-0.1 (-0.2, 0.0)	0.673
Radiotherapy				0.390
No/unknown	1457 (44.3)	1009 (43.8)	448 (45.4)	
Yes	1832 (55.7)	1294 (56.2)	538 (54.6)	
Chemotherapy				0.755
No/unknown	3271 (99.5)	2291 (99.5)	980 (99.4)	
Yes	18 (0.5)	12 (0.5)	6 (0.6)	

SEER, the Surveillance, Epidemiology, and End Results; G1, well moderately differentiated; G2, moderately differentiated; G3, poorly differentiated; G4, undifferentiated; RLN, excision lymph nodes; PLN, positive lymph nodes; NLN, negative lymph nodes; LNR, lymph node ratio; LODDS, log odds of positive lymph nodes; IQR, interquartile range.

**Table 2:** Baseline characteristics of patients for external validation cohort from China hospital database.

Variables	Total (n = 215)	%
Age, Mean ± SD	47.7 ± 12.8	
Age		
21-52	145	67.4
53-63	45	20.9
64-83	25	11.6
Sex		
Male	48	22.3
Female	167	77.7
Grade		
G1	164	76.3
G2	39	18.1
G3	10	4.7
G4	2	0.9
T		
T1	177	82.3
T2	23	10.7
T3	13	6.0
T4	2	0.9
N		
N0	173	80.5
N1	42	19.5
Stage		
I	191	88.8
II	21	9.8
III	1	0.5
IV	2	0.9
Surgery		
Thyroid tumor, adenoma, partial, lesion, single cervical lymph node dissection	51	23.7
Subtotal thyroidectomy, major thyroidectomy	35	16.3
Total thyroidectomy, radical thyroidectomy for thyroid cancer	129	60.0
RLN		
Median (IQR)	1.0 (1.0, 3.0)	
NLN		
Median (IQR)	1.0 (1.0, 2.0)	
LNR		
Median (IQR)	0.0 (0.0, 0.0)	
LODDS		
Median (IQR)	-0.5 (-0.5, -0.5)	

SD, standard deviation; G1, well moderately differentiated; G2, moderately differentiated; G3, poorly differentiated; G4, undifferentiated; RLN, excision lymph nodes; NLN, negative lymph nodes; LNR, lymph node ratio; LODDS, log odds of positive lymph nodes; IQR, interquartile range.

### Predictive model construction and validation

**Figure 2:**
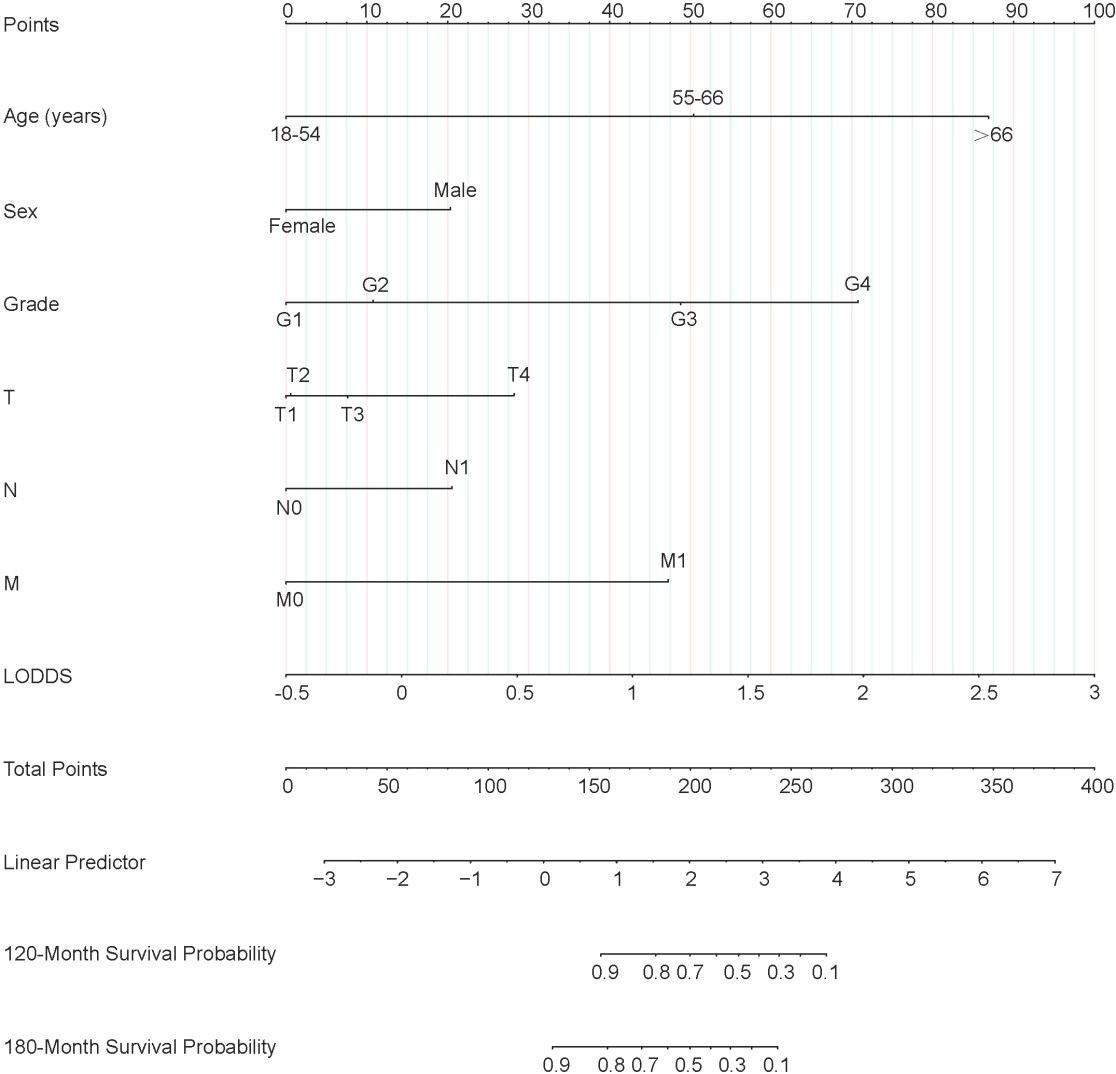
Nomogram predicting 120-month and 180-month OS of patients with PTC. OS, overall survival; PTC, papillary thyroid cancer; LODDS, log odds of positive lymph nodes.

**Figure 3:**
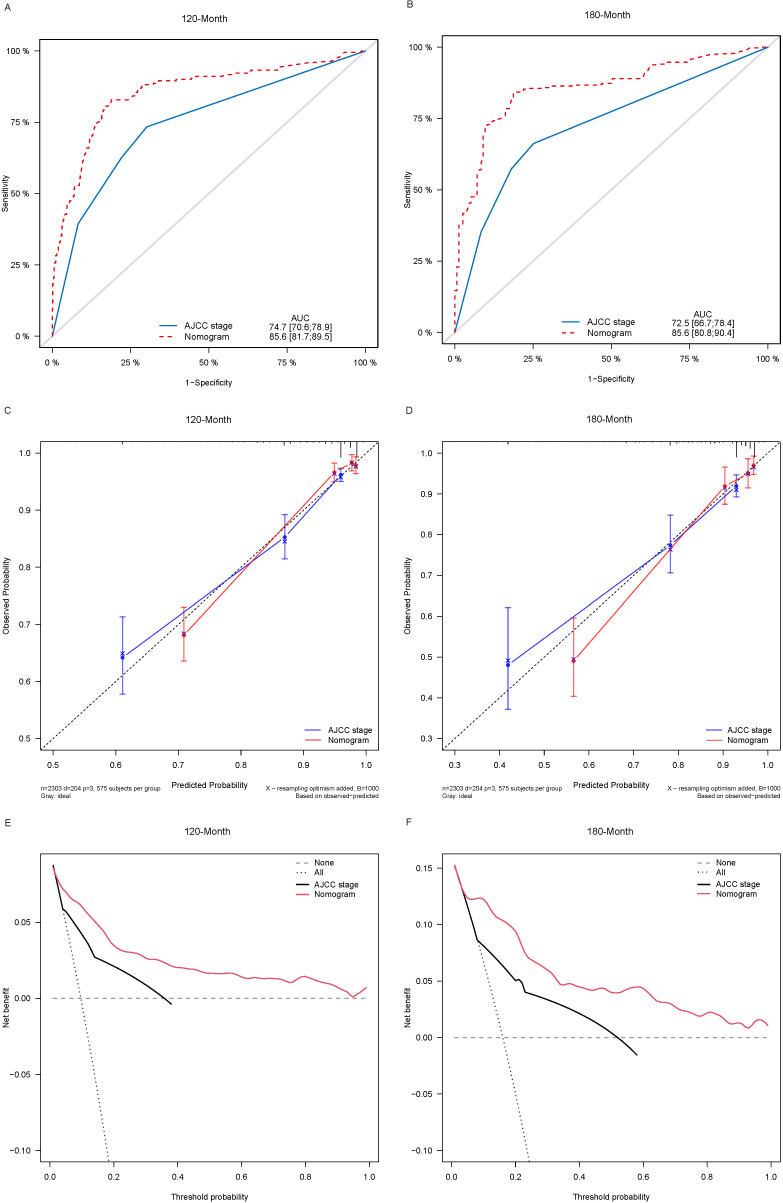
Comparison of the ROC curves **(A, B)**, calibration plots **(C, D)**, and decision curve analysis curves **(E, F)** of the two candidate models (AJCC stage and Nomogram) for 120- and 180-month OS prediction in the training cohort. ROC, receiver operating characteristic; OS, overall survival; AJCC, American Joint Committee on Cancer.

**Figure 4:**
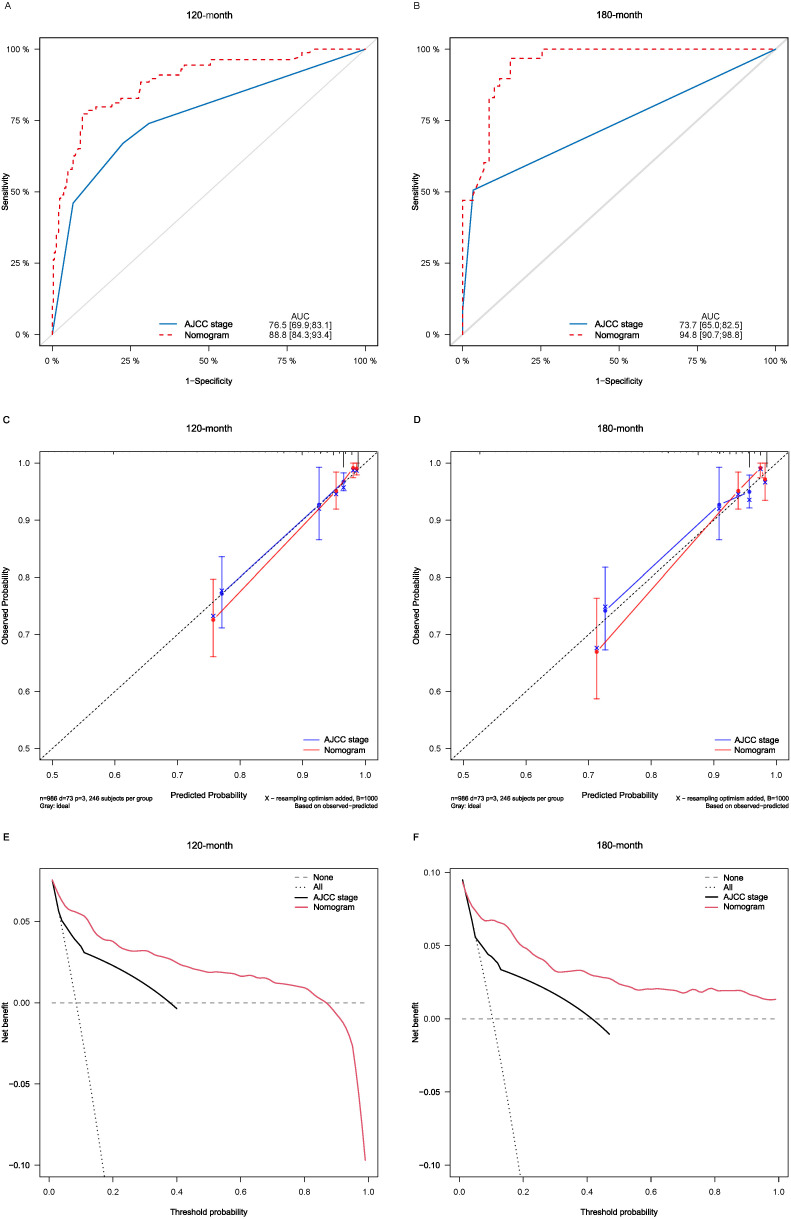
Comparison of the ROC curves **(A, B)**, calibration plots **(C, D)**, and decision curve analysis curves **(E, F)** of the two candidate models (AJCC stage and Nomogram) for 120- and 180-month OS prediction in the internal validation cohort. ROC, receiver operating characteristic; OS, overall survival; AJCC, American Joint Committee on Cancer.

**Figure 5:**
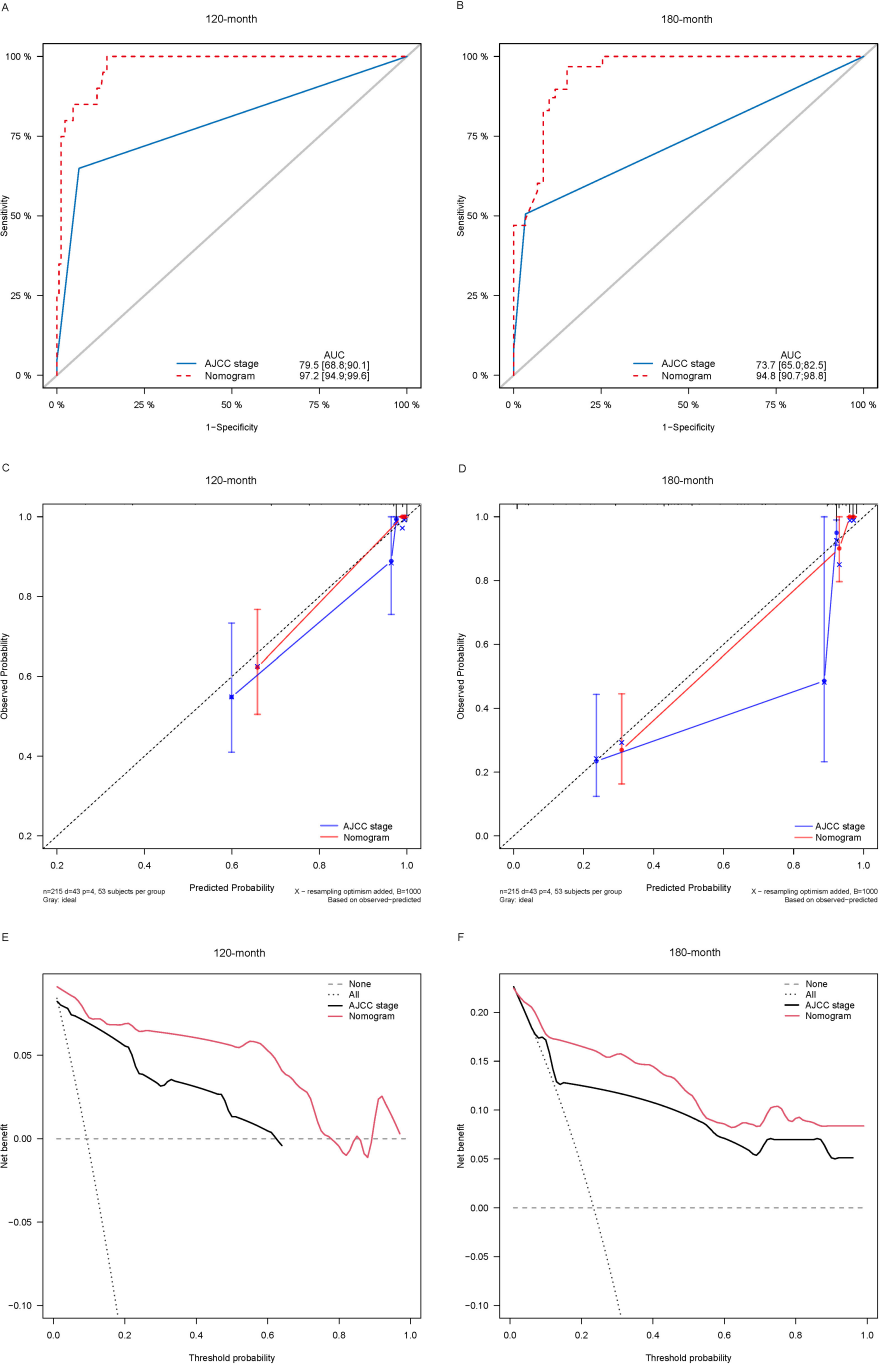
Comparison of the ROC curves **(A, B)**, calibration plots **(C, D)**, and decision curve analysis curves **(E, F)** of the two candidate models (AJCC stage and Nomogram) for 120- and 180-month OS prediction in the external validation cohort. ROC, receiver operating characteristic; OS, overall survival; AJCC, American Joint Committee on Cancer.

Lasso-multifactorial regression analysis identified age, gender, grade of differentiation, TNM staging, and LODDS as prognostic correlates of PTC (*p* < 0.05) (**Supplementary Figure S1**; **Table 3**), and nomograms were constructed to assess the probability of survival for different individuals, as detailed in **Figure 2**. The C-index of the training cohort was 0.858 (0.827-0.888), the C-index of the internal validation cohort was 0.882 (0.836-0.929), and the C-index for the external validation cohort was 0.942 (0.918-0.965). **Figure 3** compares the ROC curves of the nomogram with AJCC staging at 120 months and 180 months, respectively, and the corresponding AUC at 120 months (74.7% vs. 85.6%) and 180 months (72.5% vs. 85.6%). The AUC of the nomogram was greater than the AUC of AJCC staging for both the training cohort and validation cohort. The comparative accuracy analyses showed that IDI or NRI of the OS of the training or validation cohorts at 120 and 180 months were greater than 0, both *p* < 0.05 (**Table 4**), indicating that the predictive ability of the nomogram was superior to that of the traditional staging of AJCC. Further application of DCA to evaluate its clinical applicability based on the training and validation cohorts, and comparison of DCA of nomogram with AJCC staging showed (**Figures 3**–**5**) added more net benefit than AJCC staging for almost all threshold probabilities, which suggests that the use of nomogram for predicting the OS of radiotherapy in post-surgical PTC patients is more applicable to clinical decision-making than AJCC staging.

**Table 3:** Multivariate Cox analysis on variables for the prediction of overall survival of PTC patients.

Variable	Multivariate Cox analysis	
	HR (95%CI)	*p*-Value
Age		
18-54	1	
55-66	4.07 (2.86–5.79)	<0.001
>66	11.28 (8.16–15.59)	<0.001
Sex		
Male	1	
Female	0.52 (0.40–0.67)	<0.001
Grade		
G1	1	
G2	1.30 (0.94–1.80)	0.114
G3	4.17 (2.96–5.90)	<0.001
G4	8.29 (4.89–14.06)	<0.001
T		
T1	1	
T2	1.13 (0.77–1.65)	0.541
T3	0.95 (0.67–1.35)	0.763
T4	1.96 (1.29–2.99)	0.002
N		
N0	1	
N1	1.63 (1.11–2.39)	0.013
M		
M0	1	
M1	2.85 (1.87–4.33)	<0.001
Surgery		
Localized thyroidectomy/thyroid isthmus resection	1	
Subtotal thyroidectomy	1.52 (0.74–3.12)	0.250
Total thyroidectomy	0.70 (0.46–1.06)	0.090
PLN	1.02 (0.99–1.05)	0.178
NLN	1.00 (0.99–1.02)	0.715
LODDS	0.50 (0.27–0.92)	0.025
Chemotherapy		
No/unknown	1	
Yes	1.59 (0.80–3.16)	0.186

PTC, papillary thyroid cancer; HR, hazard ratio; CI, confidence interval; G1, well moderately differentiated; G2, moderately differentiated; G3, poorly differentiated; G4, undifferentiated; PLN, positive lymph nodes; NLN, negative lymph nodes; LODDS, log odds of positive lymph nodes.

**Table 4:** NRI and IDI of the nomogram and the AJCC staging system in survival prediction for PTC patients.

Index	Training cohort			Internal validation cohort			External validation cohort		
	Estimate	95% CI	*p*-value	Estimate	95% CI	*p*-value	Estimate	95% CI	*p*-value
NRI (vs. AJCC stage)									
120-month OS	0.465	0.357–0.561	< 0.001	0.415	0.212–0.570	< 0.001	0.779	0.561–0.899	< 0.001
180-month OS	0.407	0.160–0.662	0.020	0.377	0.130–0.577	0.006	0.730	0.573–0.866	< 0.001
IDI (vs. AJCC stage)									
120-month OS	0.191	0.148–0.238	< 0.001	0.190	0.128–0.276	< 0.001	0.329	0.228–0.508	< 0.001
180-month OS	0.182	0.095–0.267	0.018	0.205	0.132–0.299	0.002	0.462	0.377–0.581	< 0.001

NRI, net reclassification improvement; IDI, integrated discrimination improvement; AJCC, American Joint Committee on Cancer; PTC, papillary thyroid cancer; VS, Versus; CI, confidence interval; SEER, the Surveillance, Epidemiology, and End Results; OS, overall survival.

Using X-tiles software, patients were categorized into 3 subgroups based on the score calculated from the linear prediction of nomogram (**Supplementary Figure S2**): low-risk group (-1.67 ≤ linear score ≤ 0.55, 68 ≤ total score ≤ 107), medium-risk group (0.55 < linear score ≤ 2.19, 107 < total score ≤ 205) and high-risk group (2.19 < linear score ≤ 6.91, 205 < total score ≤ 378), which showed significant differences among the three risk groups by Kaplan-Maier curve analysis (**Supplementary Figure S3**).

### Clinical applications of the nomogram

**Figure 6:**
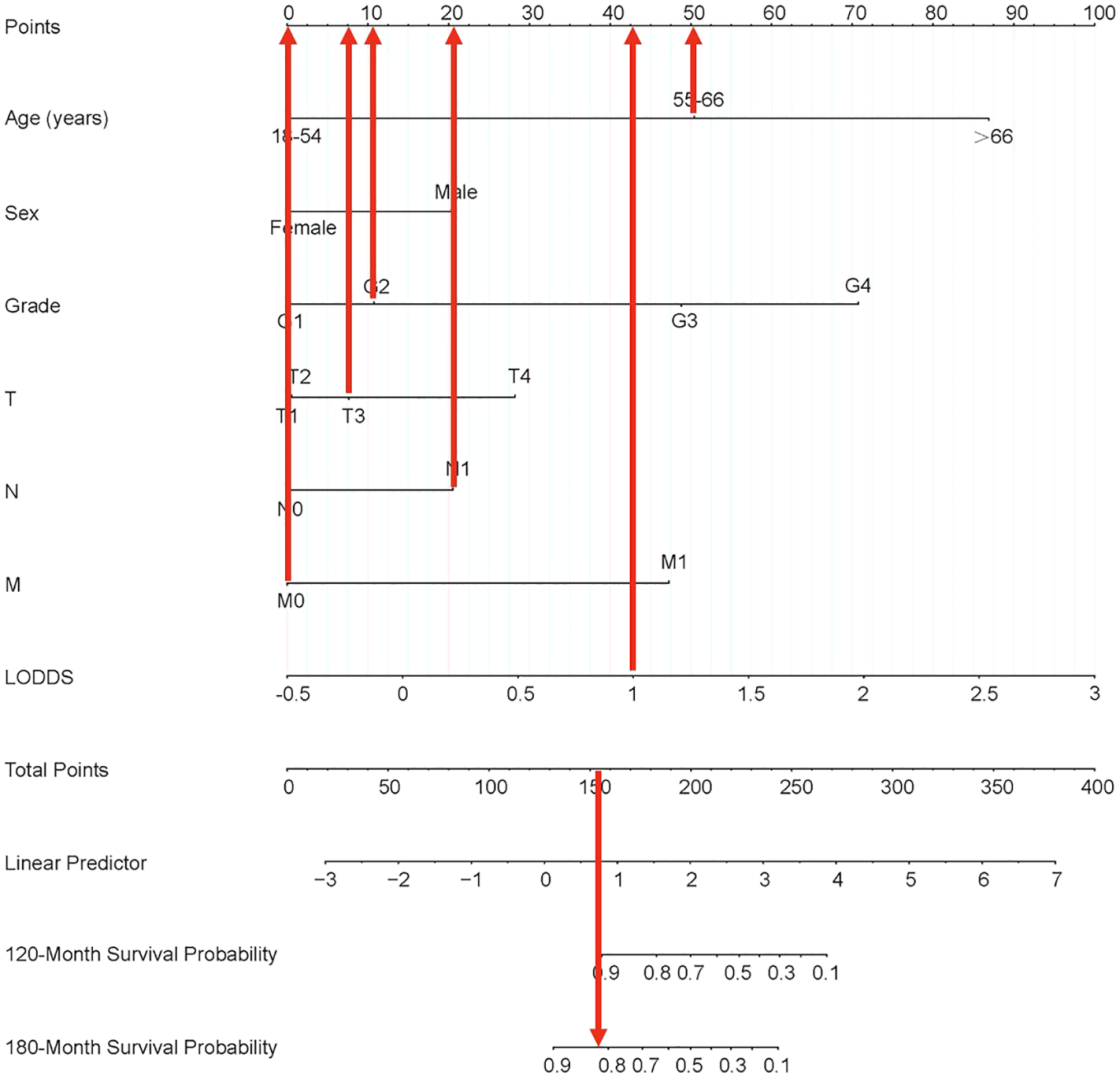
Nomogram clinical applications of nomogram predicting 120-month and 180-month OS of PTC patients. OS, overall survival; PTC, papillary thyroid cancer; LODDS, log odds of positive lymph nodes.

Each variable of the nomogram was divided equally into multiple score points, and each category of these predictor variables corresponded to a score on the counting point. The corresponding scores for each variable are summed to obtain a total score, which is positioned on the total score reference scale, and then a straight line of the survival probability scale is plotted at the bottom to estimate the OS at 120 and 180 months based on the summed total scores of the variables. For example, for a male patient aged 55-66 years, with a postoperative pathological grade of G2, T3, N1, and no distant metastases, the total score would be about 153, which corresponds to an OS of about 90% at 120 months, and an OS of about 83%-85% at 180 months (**Figure 6**).

## Discussion

PTC is one of the most common clinical subgroups of primary thyroid tumors. Accurate and rational categorization of LN status is essential for staging and prognostic assessment of patients with PTC. In this study, LODDS was shown to be independently associated with long-term clinical prognosis in postoperative PTC patients. Secondly, we constructed a visualized nomogram combining the LODDS and routinely available clinical information to predict the long-term OS of individual PTC patients. To the best of our knowledge, this study is the first to investigate the postoperative prognostic value of the LODDS for PTC based on a national and international multicenter cohort, with a relatively large sample size and a more comprehensive source of patients. It also found that LODDS is a new prognostic factor that can characterize the relationship between LN metastatic status and prognosis after surgery in PTC patients. Our nomogram using the LODDS in combination with six other variables (age, sex, grade of differentiation, and TNM stage) allowed us to assess the long-term prognosis of patients at 120 months (10 years) and 180 months (15 years). The predictive accuracy was higher than that of the conventional AJCC staging system. Prognostic analysis based on this nomogram can help clinicians to guide the postoperative rehabilitation of PTC patients, and can also more actively promote long-term follow-up.

Regarding prognostic studies in PTC, there have been retrospective studies focusing on risk factors associated with the long-term prognosis of patients. LODDS was not the only prognostic factor in the nomogram. Compared to previous studies, our results were similar to previous results where age, gender, grade of differentiation, and TNM stage were selected as prognostic factors for OS. Aamna et al. had a long-term follow-up study of 538 patients with PTC who underwent surgical intervention in a medical center over 20 years. They found that age was one of the factors associated with the prognosis of PTC (24). Luo et al. developed a nomogram prediction model based on LNM using patient data from a single center and found that LN, age, and other localized LNM in patients with PTC have been identified as the important factors affecting the prognosis of malignant tumors. The 8th edition of AJCC staging for thyroid cancer categorizes lymph node metastasis in the neck overall as N1, further subdividing metastasis to unilateral or bilateral level IV or VII lymph nodes as N1a, and metastasis to unilateral, bilateral, or contralateral levels I-IV or retropharyngeal lymph nodes as N1b, without consideration of the statistical count of metastatic lymph nodes. However, as a new prognostic indicator based on the LN ratio, LODDS can be used as an independent prognostic factor in many cancer types, whether they are common malignant tumors such as gastric cancer (4), colorectal cancer (25), and endometrial cancer (26); or rare malignant tumors such as neuroendocrine cancer of the lungs (16), pancreatic cancer (5), and bile duct cancer (17). This makes our study theoretically grounded and inspired to also explore the role of LODDS in the prognosis of thyroid cancer. Due to the favorable prognosis of thyroid cancer, with a near 100% short-term survival rate, it has prompted contemplation regarding long-term survival in our research endeavors.

In recent years, there have also been studies examining the relationship between the number of LN and thyroid prognosis by combining data from publicly available databases and other medical centers. Wang et al. based on the SEER database and patient information from multiple centers, made improvements to the eighth edition of AJCC’s N staging by analyzing the number of LN in medullary cancer of the thyroid, which was similar to the way this study was conducted (27). However, this was performed only for medullary cancer with LN, and there is still a lack of studies related to LN for papillary cancer.

At this stage, the development of nomograms to assess the prognosis of patients with PTC has been widely studied. Garo et al. conducted a systematic evaluation and analysis of previously established nomogram related to PTC (28) and found only one nomogram model for OS, which had an accuracy value of only 0.77, and it was not validated using an external validation cohort. Our study bridges the gaping problem that existed in previous studies. Currently, there remains a deficiency in the identification of novel prognostic variables associated with prognosis when utilizing the nomogram system to assess the prognosis of patients with PTC. While existing staging categorizes PTC, its application by clinicians to evaluate individual patient cases is overly generalized. The AJCC staging system exhibits relatively lower accuracy in predicting long-term prognosis, necessitating further exploration for more precise prognostic methodologies.

Although the LODDS based nomogram constructed in this study showed high accuracy in predicting the prognosis of patients with PTC, there are still some limitations. First, although the SEER database contains a large volume of data, it lacks detailed patient medical histories and data on diagnostic procedures, such as past medical history, thyroid pathological molecular markers, radiotherapy dosage, and specific drugs and dosages used in chemotherapy. Second, patients’ genetic information is not available in databases. More and more studies have shown that genetic mutations (e.g., BRAF V600E, TERT promoter mutations) (29) and epigenetic alterations (e.g., DNA methylation, noncoding RNA expression) (30) play important roles in the occurrence, development, and prognosis of PTC. For example, BRAF V600E mutation is strongly associated with aggressiveness and poor prognosis of PTC, while the expression levels of certain miRNAs may be associated with the risk of lymph node metastasis and recurrence (15). Our study was mainly based on clinicopathologic factors and did not include genetic and epigenetic factors. In addition, single-center externally validated cases lacked pooling of hematology results and imaging results, and require further expansion and optimization both in terms of population size and source prevalence, which gives us further direction for future research efforts.

With the current study, we found that the LODDS was associated with prognosis in PTC and had a more significant prognostic correlation than other LN staging. The new nomogram also showed better prognostic assessment value in predicting postoperative OS in patients with PTC than the AJCC staging system counted in the SEER database. Patients stratified into low-risk, medium-risk, and high-risk groups based on their LODDS scores display significant differences in long-term OS according to Kaplan-Meier analysis, thereby facilitating better risk stratification for prognosis. In summary, our developed predictive LODDS staging system outperforms traditional staging systems, aiding clinicians in identifying high-risk populations and accurately assessing long-term prognosis.

## Conclusions

In conclusion, we identified factors that influence postoperative survival in patients with PTC. For the first time, our population-based and aforementioned factor-based study established nomograms for PTC patients undergoing surgery that predicted patients’ 120-month survival and 180-month survival, as well as possible prognosis in the long-term. The established nomograms objectively showed good accuracy and clinical applicability and provided prognostic information of limited value, providing an individualized, long-term clinical prognostic reference for the treatment of PTC patients.

## Data Availability

The raw data supporting the conclusions of this article will be made available by the authors, without undue reservation.

## Supplementary material

Supplementary Figure 1 To further narrow the range of variables involved in the regression analysis, the parameters were adjusted by 10-fold cross-validation, and established using the least absolute shrinkage and selection operator (LASSO) in the Cox model in the training cohort **(A).** Combining the distribution of LASSO coefficients for eleven variables (age, sex, grade, T stage, N stage, M stage, surgery, PLN, NLN, LODDS and chemotherapy) in PTC patients, an optimal lambda filter was used to generate six variables (age, sex, grade, T stage, N stage, M stage, and LODDS) with non-zero coefficients **(B)**. PLN, positive lymph nodes; NLN, negative lymph nodes; LODDS, log odds of positive lymph nodes; PTC, papillary thyroid cancer.

Supplementary Figure 2 X-tile cutoff analysis.

Supplementary Figure 3 Risk stratification Kaplan-Meier overall survival curve for patients in the training cohort after completion of cut-off analysis at X-tile.
