# Supplementary figures and images for: Construction and validation of novel nomograms based on the log odds of positive lymph nodes to predict the prognosis of papillary thyroid cancer: a retrospective cohort study
Source: Front Endocrinol (Lausanne). 2025 Mar 7;16:1411426. doi: 10.3389/fendo.2025.1411426 (PMC11925767; doi:10.3389/fendo.2025.1411426)

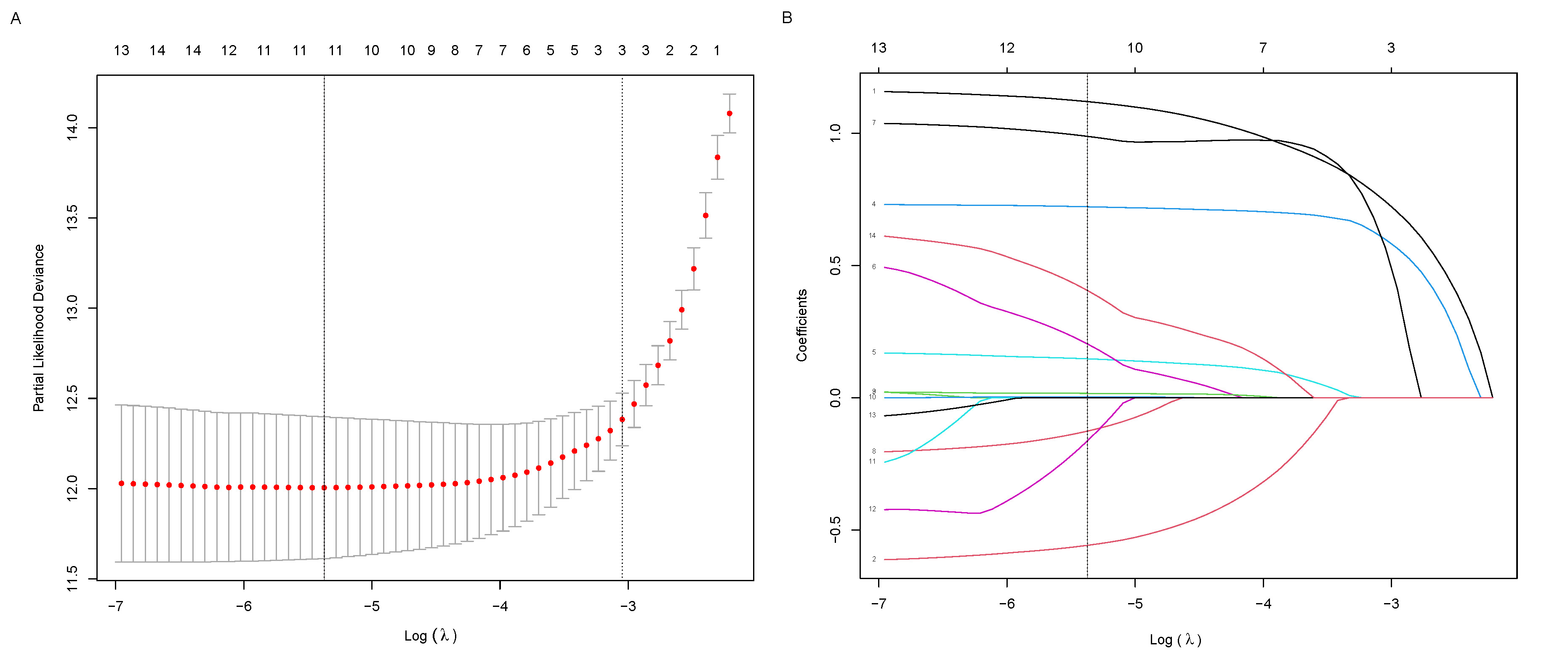

Supplement: Supplementary Figure 1 — To further narrow the range of variables involved in the regression analysis, the parameters were adjusted by 10-fold cross-validation, and established using the least absolute shrinkage and selection operator (LASSO) in the Cox model in the training cohort (A). Combining the distribution of LASSO coefficients for eleven variables (age, sex, grade, T stage, N stage, M stage, surgery, PLN, NLN, LODDS and chemotherapy) in PTC patients, an optimal lambda filter was used to generate six variables (age, sex, grade, T stage, N stage, M stage, and LODDS) with non-zero coefficients (B). PLN, positive lymph nodes; NLN, negative lymph nodes; LODDS, log odds of positive lymph nodes; PTC, papillary thyroid cancer. [file Image1.jpeg]

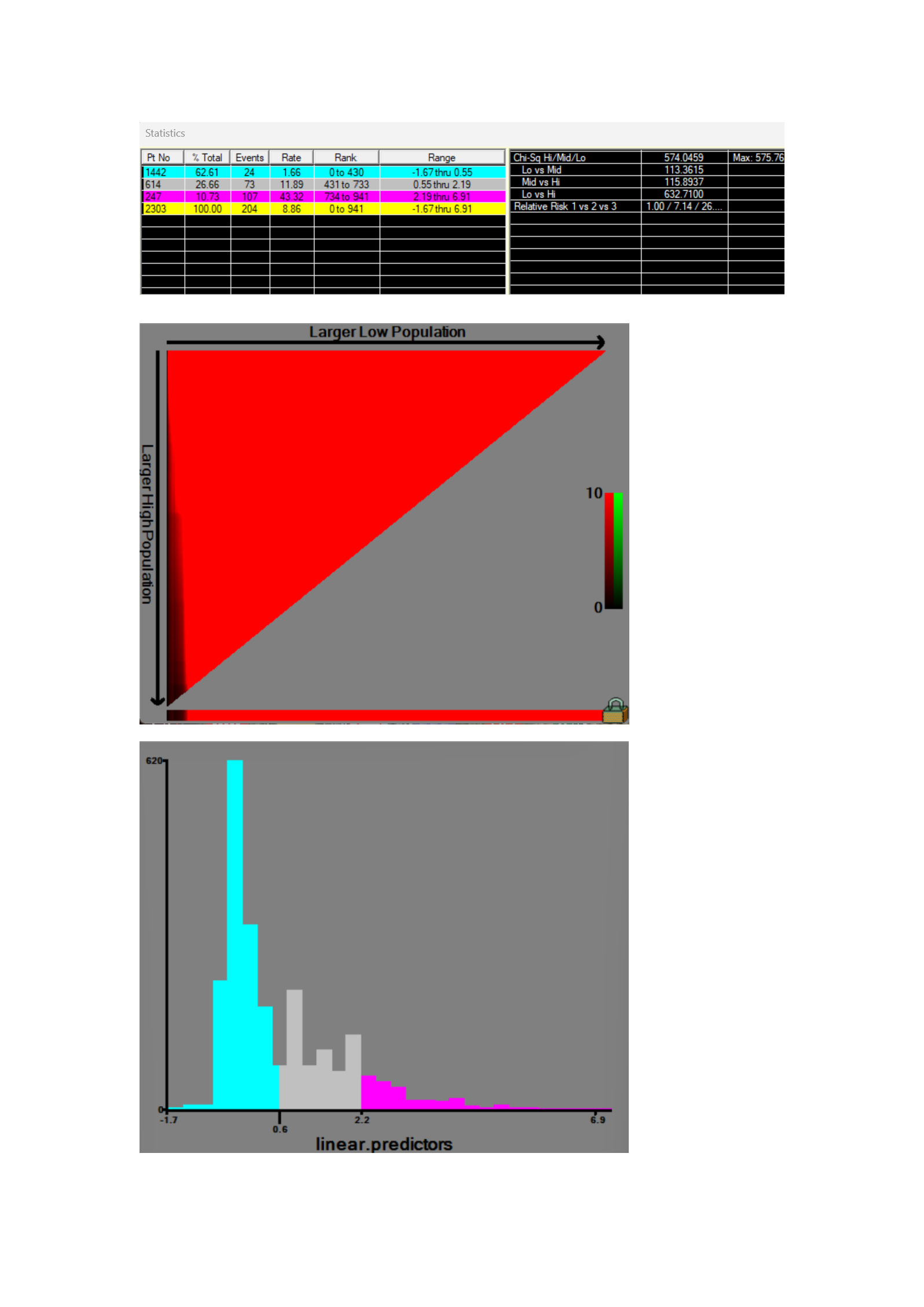

Supplement: Supplementary Figure 2 — X-tile cutoff analysis. [file Image2.jpeg]

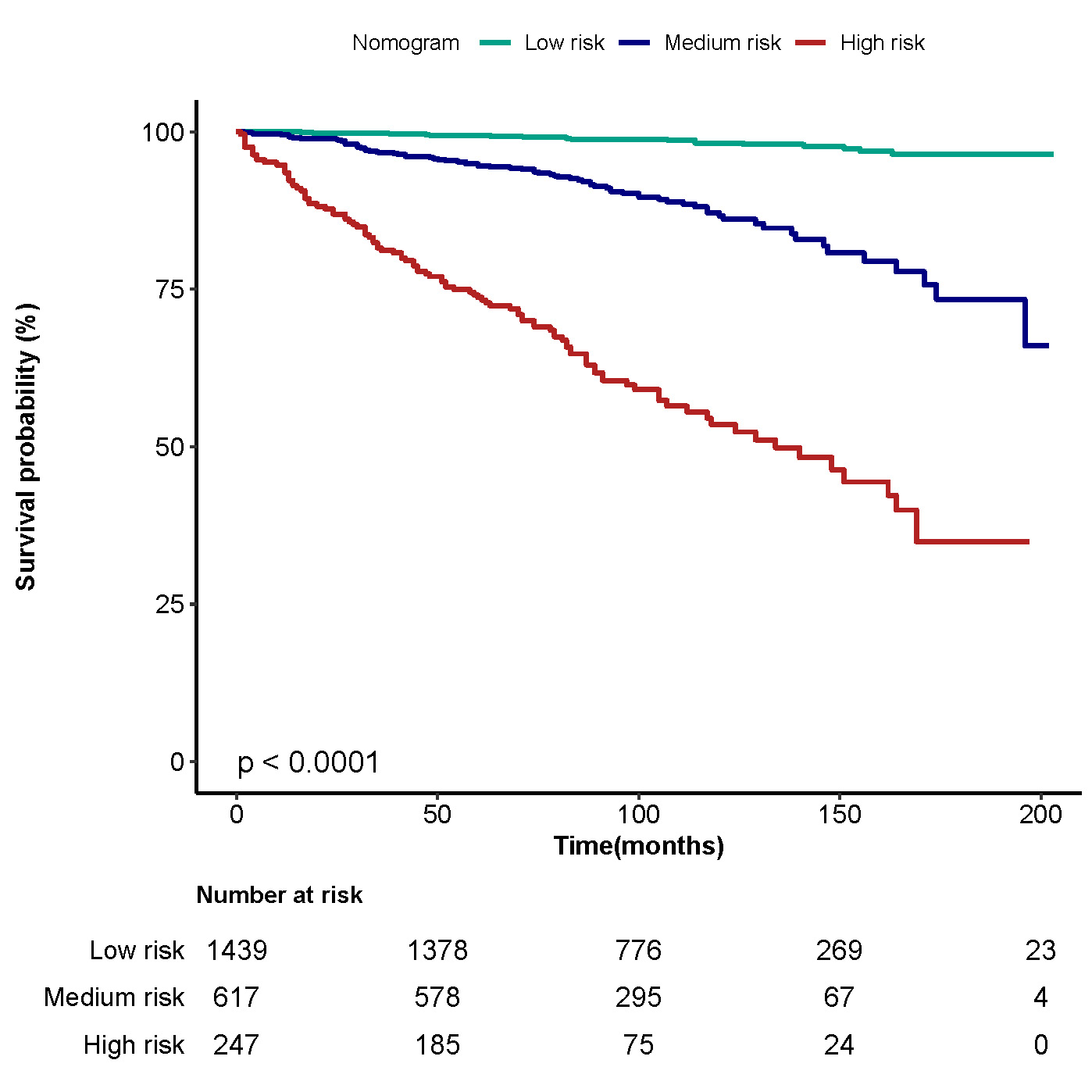

Supplement: Supplementary Figure 3 — Risk stratification Kaplan-Meier overall survival curve for patients in the training cohort after completion of cut-off analysis at X-tile. [file Image3.jpeg]
